# A Systematic Review of Modeling Platforms for Atrioventricular Valves in Atrioventricular Septal Defects

**DOI:** 10.1007/s12265-026-10799-z

**Published:** 2026-06-18

**Authors:** Chen Chia Wang, Murray Marx, Eric Barth, Antonio D’Amore, Haoxiang Luo, Garrett Coyan

**Affiliations:** 1 Department of Cardiac Surgery, Vanderbilt University Medical Center, Nashville, TN USA; 2 Department of Mechanical Engineering, Vanderbilt University, Nashville, TN USA; 3 Department of Bioengineering, University of Pittsburgh, Pittsburgh, PA USA; 4 Department of Surgery, University of Pittsburgh Medical Center, Pittsburgh, PA USA; 5 Department of Biomedical Engineering, Vanderbilt University, Nashville, TN USA; 6 Division of Pediatric Cardiac Surgery, Vanderbilt University Medical Center, Nashville, TN 37232 USA

**Keywords:** Atrioventricular valve, Heart valve, Atrioventricular septal defect, In silico, In vitro, In vivo, Model, 3D print, Flow loop, Pulse duplicator

## Abstract

Given the high rate of re-intervention in patients with atrioventricular septal defects (AVSD), there is a continuing need to accurately model the complex valve anatomy and surgical repair strategies. This systematic review examined the current scientific landscape for modeling platforms designed to study abnormal congenital heart valves that are used or could be used for AVSD investigation. Of 1,050 sources screened, 47 were included (14 in silico; 26 in vitro; 7 in vivo). In silico models studying valve pathologies have advanced in recent years, but few targeted AVSD valves. In vitro platforms allow comparisons of repair techniques, but have yet to be applied for AVSD valve repair. In vivo studies could offer physiologically accurate platforms, but face challenges such as replicating AVSD pathologies. Multidisciplinary approaches to creating high-fidelity, reproducible, physiologically accurate AVSD valve models can transform the understanding of AVSD valve properties and repair strategies.

**Supplementary Information:**

The online version contains supplementary material available at 10.1007/s12265-026-10799-z.

## Introduction

Atrioventricular septal defects (AVSD) are a result of abnormal development of the endocardial cushions in the early embryonic period [1]. AVSD has an incidence of 0.2–0.3 per 1000 live births, and accounts for 4–5% of congenital heart diseases [2, 3]. Abnormal atrioventricular (AV) valves in patients with AVSD can range in complexity from a partial AVSD, consisting of a large atrial communication with a cleft malformation of the left-sided AV valve, to complete AVSDs that demonstrate both atrial and ventricular communication with complex left and right AV valve abnormalities [4]. 

AVSDs undergo operative repair in the first several months to years of life, depending on symptoms and morphological subtypes. While immediate post-operative mortality has improved over the years, there remains significant risk (> 15%) of reoperation within 10 years of repair [5]. Currently, the status quo of surgical techniques being applied to AV valve repair in patients with AVSD is based on historical experiences of specific surgical approaches to AV valve repair [6]. Repair strategies also vary based on specific valve morphology to some degree, but remain varied from center to center and surgeon to surgeon. Importantly, significant recurrent left AV valve regurgitation (LAVVR) occurs in upwards of 20% of patients, and there are few studies that consistently identify mechanisms for specific repair failure in these patients [7]. 

A central gap in current AVSD repair is that no reliable scientific models exist to rigorously test and compare various approaches to AV valve repair in particular patient-specific anatomical circumstances. Given that there is a wide spectrum of AV valve functional presentation among patients with AVSDs, a more rigorous and complete approach to understanding AV valve structural mechanics and function in relation to possible effects from surgical intervention would help reduce the current high rates of residual AV valve dysfunction which leads directly to patient harm. This review focuses on current modelling platforms designed to study abnormal AV valves seen in AVSD and further relates to the utility in surgical planning or technique refinement.

## Methods

### Search Strategy

This review was performed in accordance with the Preferred Reporting Items for Systematic Reviews and Meta-Analyses, extension for Scoping Reviews (PRISMA-ScR, Supplemental Document 1). Publications and book chapters describing in silico, in vitro, or in vivo modelling platforms for studying congenital heart diseases were searched on PubMed, Scopus, and Embase. No year restrictions were applied to our search. This search took place on August 21, 2025. Inclusive keywords were identified and utilized in all three databases according to specific database search algorithms to optimize article identification (Supplementary Document 2).

### Selection of Sources

Sources were selected based on relevance to our predetermined topic of modeling platforms to study congenital heart defects, specifically AVSDs. Therefore, all clinical cohort studies without the use of simulation-based interventions were excluded. Studies focused on extracardiac anatomy (such as aorta) or non-congenital heart defects were also excluded. While we aimed to focus on modelling platforms to study AVSD, studies examining other forms of congenital heart defects were included to demonstrate the potential of these platforms given the small number of specific targeted AVSD platforms. As this was a descriptive review, no specific data charting protocol, appraisal of evidence, or synthesis of results were applicable.

## Results

**Fig. 1:**
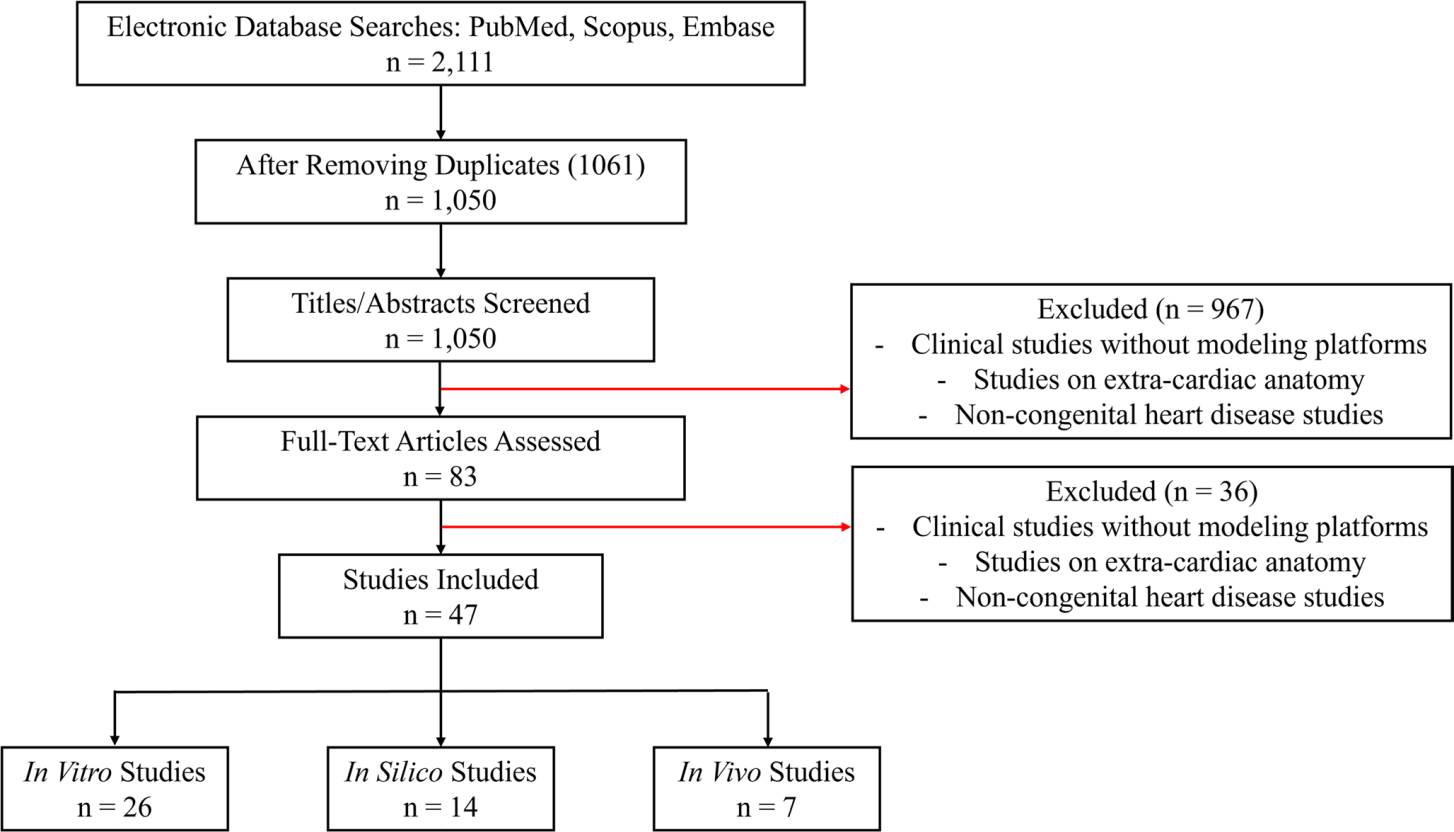
Flowsheet of search results and study inclusion/exclusion criteria

**Fig. 2:**
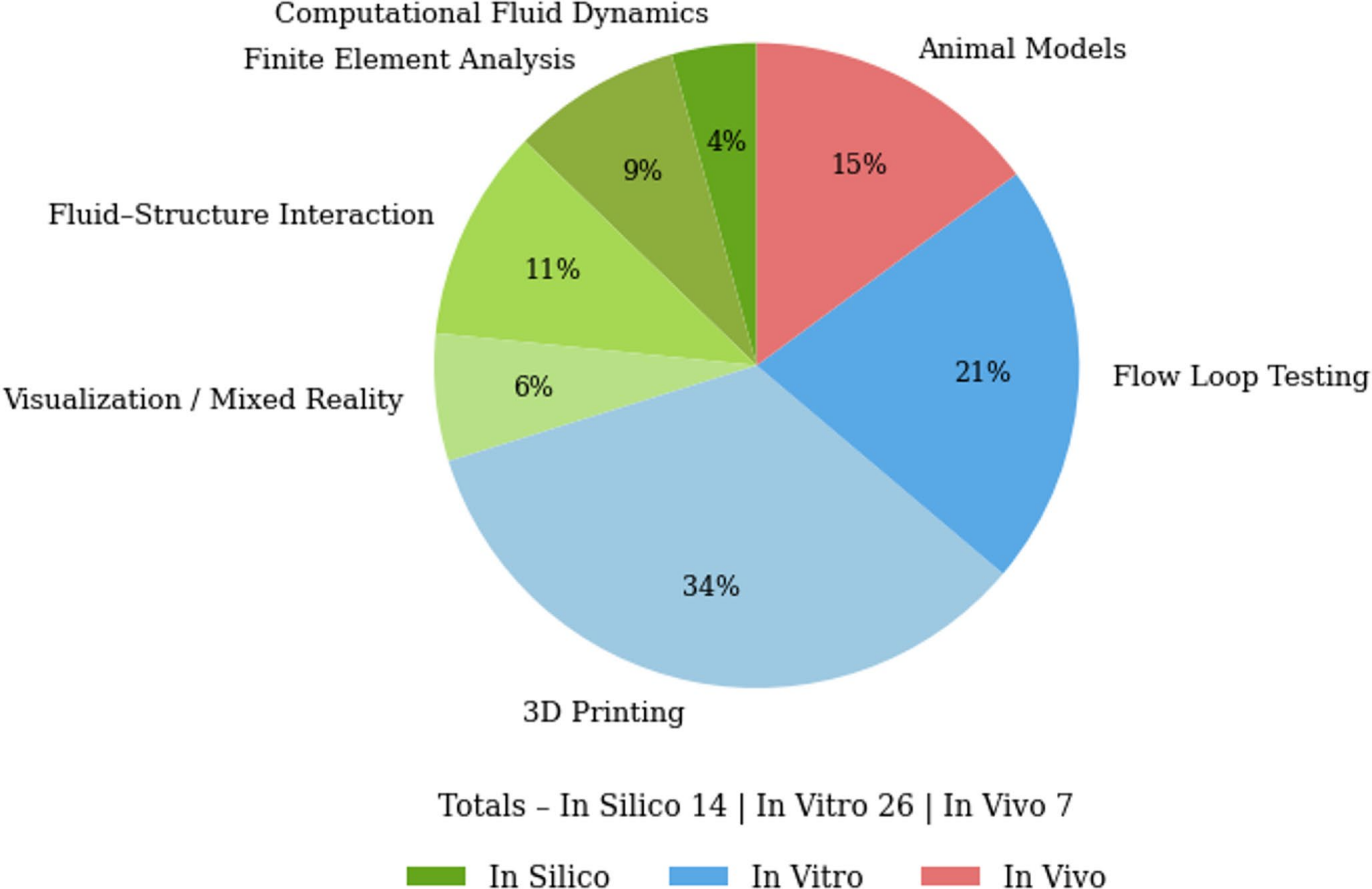
Schematic breakdown of final articles included in the review, stratified by type and subtype of modeling platform

A total of 2,111 sources resulted from our search strategy across PubMed, Scopus, and Embase, with 1,050 sources after removing duplicates (Fig. 1). All 1,050 sources were screened via title and/or abstract, from which 83 sources were deemed eligible for full-text assessment. After full-text assessment, 47 studies met the inclusion criteria, with 14 studies describing in silico modeling, 26 studies describing in vitro modeling, and 7 studies describing in vivo modeling for congenital heart defects (Fig. 2).

## In silico Models

As 3D data from computed tomography (CT), magnetic resonance imaging (MRI), and echocardiography is becoming more widely available, computational models that can interpret this data hold great potential. For example, Jones et al. was able to build a predictive model associating preoperative 3D findings on MRI and echocardiography with successful biventricular repair for unbalanced complete AVSD [8]. However, greater potential lies in the simulation capabilities that make it possible to study the complex hemodynamics and biomechanics of diseased heart valves in a controlled, customizable setting. Broadly, computational fluid dynamics, finite element analysis, and fluid-structure interaction are three available modeling techniques applicable to studying abnormal AV valves.

### Hemodynamics Modeling

To study the hemodynamics associated with complex valves, patient-specific 3D imaging data can be used for the virtual geometric reconstruction of the cardiac anatomy relevant to the valve of interest [9]. Computational fluid dynamics is a modeling approach that simulated flow when the 3D geometry and boundary conditions are set. This technique has been applied in a study by An et al. simulating blood flow patterns in bicuspid aortic valves [10]. Using patient-specific CT angiography and echocardiographic data to build the geometric constraints of the bicuspid valve and aorta, they were able to simulate the three-dimensional flow velocity field, vortex pattern, and wall shear stress. They were also able to demonstrate an improvement in abnormal blood flow patterns and wall shear stress after transcatheter aortic valve replacement. While this technique has been used for pathologic heart valve modeling in general, it has not yet been applied more broadly to abnormal valves in AVSD.

### Biomechanical Modeling

Other in silico modeling studies focus on the biomechanics of the heart valve, such as leaflet motion and ventricular geometry. Finite-element analysis is a method of simulating structural deformations when provided data on material properties (e.g., tissue stiffness) and external forces (e.g., pressure load), and is a useful tool to study these. This has been used to study leaflet motion abnormalities in an aortic and mitral regurgitation model when customizing the left ventricular geometry to represent congenital dilated cardiomyopathy [11]. Nappi et al. used finite-element analysis to study mechanical stresses at the root and leaflets of the pulmonary autograft in the aortic position after Ross operation [12]. This in silico model provided insight into the increased risk of mechanical deformation with an increased length of the conduit, which could potentially translate to practical, clinically relevant findings. Another study investigating annuloplasty ring sizes in bicuspid aortic valves used finite-element analysis to demonstrate that small ring sizes are associated with increased leaflet coaptation area and decreased stress at the coaptation zone [13]. The study also incorporated computational fluid dynamics to show that decreased ring sizes led to increased peak blood flow and wall shear. Combined, they were able to use in silico modeling to establish upper and lower limits for optimal annuloplasty diameters for bicuspid aortic valve regurgitation repair. Another group used finite-element analysis to simulate the biomechanics of a novel valve stent versus that of a traditional valve stent for bicuspid aortic valve replacement, and demonstrated the potential for in silico models to help design and study novel valve stents [14]. Similarly to before, this technique has been used for pathologic heart valve modeling in general but not yet for abnormal valves in AVSD.

### Coupled Hemodynamic and Biomechanical Modeling

Lastly, fluid-structure interactions is another simulation modality that integrates both hemodynamic and biomechanical modeling of heart valves. In this technique, the geometric constraints are given as input about information of the flow domain, the solid-structure domain, and their interface. In the simulation fluid dynamics and structural mechanics are solved in a coupled fashion: the fluid applies external forces (i.e., pressure and shear stress) on the structure at the interface, and the structure responds to the load and deforms, whose displacements changes the fluid domain geometry as well as the boundary velocity, thus altering the flow behavior subsequently. Using patient-specific imaging data, various studies have used fluid-structure interactions to provide insight into the biomechanics (e.g., stresses on the leaflets, leaflet movement/deformation, and leaflet coaptation) and hemodynamics (e.g., flow velocity, vortex pattern, wall shear stress) of bicuspid aortic valves [15–17]. Importantly, Helbock et al. was able to use fluid-structure interaction to design a novel asymmetric aortic valve specifically for bicuspid aortic valve replacements, and simulate the changes in hemodynamics when using the novel valve compared to current commercial valves [18]. A Stanford group was able to apply in silico modeling using fluid-structure interactions to simulate the effects of bicuspidization of severe congenital aortic valve abnormalities [19]. Specifically, they were able to identify an optimal range of free edge lengths to avoid stenosis and excessive leaflet billowing. Once again, this technique has not yet been broadly applied to the abnormal valves seen in AVSD patients.

### Visualization

Lastly, in silico modeling also holds great clinical potential, especially for visualization of complex congenital heart defects for education and planning purposes. A study led by the Children’s Hospital of Philadelphia created a novel method to visualize and quantify complex valve structures in complete AV canals using 3D echocardiogram data [20]. Importantly, they were able to create separate models based on various timepoints of the cardiac cycle to provide insight on leaflet, papillary muscle, and annulus changes throughout systole and diastole. Furthermore, they were able to identify characteristics of the original canal annulus that portend LAVVR after repair. Another clinical application of computational modeling was demonstrated by Ponzoni et al., where CT and MRI data were used to create a mixed reality tool to assist in preoperative planning and intraoperative guidance for complex congenital heart defect repairs [21]. They were able to create mixed reality simulations of 5 different congenital heart defect repair scenarios, and found that 3D immersive simulations improved understanding of the anatomical relationships and allowed for preoperative planning to identify potential surgical strategies. These models have not been rigorously adapted to test or compare results of potential surgical interventions on AVSD.

## In vitro Models

In vitro models to study AVSD valves fall under two general categories: 3D printing of congenital heart models and flow loop testing of heart valves. 3D printing of congenital heart models is useful for anatomical visualization, education, and surgical planning/simulation, but does not provide information on the hemodynamics and biomechanics of the valves before or after proposed repair. Flow loop testing could provide insight into the fluid and structural dynamics of the valve, and can facilitate the study of different repair strategies while maintaining replicability and control over the valve testing environment, provided a suitable valve material for simulation is identified.

### 3D Printing

The majority of current in vitro modeling efforts for AVSD and congenital heart disease in general are within the 3D printing space. Several studies have shown that 3D heart models created with patient data were accurate. Perens et al. demonstrated that MRI and CT images from patients needing biventricular repair could be accurately converted to a 3D printed model using a free software and basic printer [22]. Another study created 3D models from echocardiographic data from eight patients with ventricular septal defects and one with periprosthetic aortic valve leaks, and found an accuracy of <1 mm error in the 3D models [23]. 

As the accuracy of 3D printed models using patient-specific data has been validated, multiple reports also highlighted the various use cases and benefits of additive manufacturing. Lobbestael et al. reported the use of CT images to create a 3D model that helped preoperative planning for a patient with complete AVSD, transposition of the great arteries, sub-pulmonary stenosis, L-looped ventricles, hypoplastic right ventricle, and a distant aorta arising from the right ventricle [24]. Similar benefits in facilitating diagnosis and preoperative planning have been shown in various clinical presentations such as two patients with concordant AV connections to L-looped ventricles in the presence of situs solitus, and another patient with heterotaxy syndrome, dextrocardia, double-outlet right ventricle, and complex pulmonary valve stenosis [25, 26]. An institutional case series highlighted five cases where 3D printing facilitated pre-operative planning in the setting of multiple prior operations and complications, and importantly helped identify feasible repair strategies in a patient recommended for palliative care and another patient recommended for combined heart-lung transplant by other institutions [27]. Similarly, an institutional experience of 3D printing for congenital heart disease in Turkey found that the in vitro models helped refine the surgical plan and better identify risk structures before the operation [28]. Another study by Kolcz et al. compared patients undergoing surgical unifocalization of major aortopulmonary collateral arteries (MAPCAs) with traditional preoperative planning versus others additionally assessed using 3D printed models and virtual reality [29]. They found that the patients with virtual reality and 3D printed models were used had lower operative times and intraoperative complications, as well as better parent satisfaction with the care and improved comprehension of their child’s anatomy.

Another benefit of 3D modeling of congenital heart disease is in education and training. The University of Toronto group has developed a hands-on surgical training platform using a rubberlike material to simulate surgical procedures on 3D models based on patient-specific imaging data [30]. While the difference between the properties of the material and human tissue was a limitation, most surgeons/trainees who participated found the quality acceptable for the purpose of surgical practice. Another group from Japan developed accurate 3D models using flexible polyurethane that mimic the elasticity of a child’s heart for surgeons to simulate the planned operation, and the surgeons found that the model was essential in the successful repair of 13/19 (68%) patients [31]. Lastly, Ilina et al. created patient-specific silicone valve models for surgeons to practice valve repair strategies, which were well received and considered to be valuable for valve surgery training [32]. 

In addition to these case reports and single-center experiences, the benefits of 3D printing in congenital heart disease have also been reiterated in several reviews [33–37]. This includes benefits in diagnosis, surgical training, and surgical planning for congenital heart diseases with complex anatomy [33, 35]. Specifically, 3D models have already been developed and used to study valvular heart disease, including interventions on all four cardiac valves, as well as other structural heart interventions such as left atrial appendage occlusion [34]. The Radiological Society of North America’s 3D Printing Special Interest Group also provided a consensus appropriateness ratings document in 2024 summarizing the clinical standards of 3D printing for pediatric congenital heart disease scenarios [37]. Specifically, they deemed 3D printing to be usually appropriate and advantageous in representing/extending medical imaging data in scenarios of complex atrial septal defects, complex ventricular septal defects, and unbalanced AV canals.

However, 3D models have limited utility beyond basic visualization and educational purposes as they are rigid anatomical replicas without functional utility. 3D printed models are unable to replicate the opening, closing, and leaflet coaptation required to study the actual hemodynamics and biomechanics of AVSD valves. Given their lack of functionality, 3D printed models are currently only used for surgical training or general repair visualization and not designed to allow for robust testing and comparisons of different valve repair strategies. Overall, 3D printing represents a viable option to create visualization models of AVSD valves for education and basic planning, but has limited utility in the functional flow dynamics testing required to enhance our understanding of AVSD valves.

### Flow Loop Testing

In vitro functional hemodynamic platforms for benchtop studies of heart valves are generally designed as a valve within a flow loop, where an actuation device generates pulsatile flow through the valve of interest. While components of the flow loop differ based on the exact needs of each study, most designs include a ventricle (pump), a valve holder, compliance chamber, and an atrium/venous reservoir. These platforms are also generally designed to include flow and pressure sensors to capture hemodynamic data, and to be compatible with imaging modalities such as videography or echocardiography. In vitro platforms to study valves have been more widely used to study adult valve pathologies, but important progress has been made in the congenital valve space as well.

Various groups have used an in vitro pulse duplicator platform to study bicuspid aortic valves. In 2014, Juraszek et al. studied porcine bicuspid aortic valves in a flow loop and found altered hemodynamic patterns downstream of the valve that may contribute to aneurysmal development in patients with bicuspid aortic valves [38]. More recently, the Stanford group presented a report studying a rare porcine type 0 lateral bicuspid aortic valve [39]. They were able to model the valvular hemodynamics at baseline, in an aortic aneurysm setting, and after valve-sparing root replacement to demonstrate the potential benefits of valve-sparing root replacement in cases of bicuspid aortic valve regurgitation. The same team conducted another study where they created porcine bicuspid aortic valves with left-right cusp fusion—the most common phenotype of bicuspid aortic valves—and demonstrated reduced regurgitant fraction after repair using their in vitro left heart simulator [40]. Another report studied a rare porcine quadricuspid aortic valve, and found altered hemodynamics and improper coaptation that could lead to valve failure over time [41]. Another study by Anam et al. selected three patients with bicuspid aortic valves that had paravalvular leakage after transcatheter aortic valve replacement, and used patient-specific data to create 3D models of the valves that they tested in a flow loop before and after transcatheter aortic valve replacement [42]. Importantly, the paravalvular leakage degree and locations in the in vitro model matched the clinical data, highlighting the potential of in vitro platforms to simulate interventions to guide operative planning in heart valve disease. Furthermore, groups have also used in vitro modeling platforms to compare surgical techniques in bicuspid aortic valve repair. Romagnoni et al. tested porcine bicuspid valves repaired with either 120° (native) or 180° (newly proposed technique, but not validated) between the two repositioned commissures [43]. They were able to show that 180° commissural reposition does not improve transvalvular gradients at rest, but results in lower gradients under mild exercise conditions. Similarly, Arimura et al. used a flow loop to study sinus plication during bicuspid aortic valve repair, another new and promising technique [44]. They were able to demonstrate that sinus plication improved valve opening, which significantly reduced the transvalvular gradient and regurgitant fraction.

Several groups have also investigated other congenital valve diseases using an in vitro modeling platform. Boone et al. reported an effort to create tricuspid valves in hypoplastic left heart syndrome using patient-derived 3D echocardiography, and using a flow loop to explore the unique anatomical and hemodynamic relationships in children with hypoplastic left heart syndrome [45]. Kidambi et al. created customized valve annuli using imaging data from patients with hypoplastic left heart syndrome, and compared tricuspid valvular biomechanics in cases of annular dilation and/or leaflet tethering [46]. They found that annular dilation was associated with increased forces on the septal leaflet, whereas leaflet tethering increased forces on all three leaflets. Choi et al. studied the bicuspidization repair of congenitally diseased aortic valves, with a specific focus on the free-edge length to aortic diameter ratio [47]. They found that a ratio of 1.57 resulted in the lowest transvalvular gradient, regurgitant fraction, and largest orifice area compared to other ratios, which are important insights that can guide future surgical repairs.

Overall, in vitro platforms using a flow loop to study AV valves in AVSD represent a frontier that could drastically improve the current understanding of the underlying mechanics of congenital valve defects, and offer vital information to guide operative repairs, provided an appropriate modeling substrate could be identified.

## In vivo Models

In vivo models represent the highest fidelity platform to study valves at near- physiological conditions, but are limited by the resource-intensive nature and difficulty in replicating animal models with congenital heart defects. The complex study environments in a living animal also make it challenging to create reliable and replicable valve research platforms. Despite these obstacles, in vivo modelling platforms to study abnormal AV valves have been developed. Broadly speaking, in vivo models to study the genetic and developmental causes of congenital heart defects are done in small animals, whereas models to study novel devices or techniques for treatment of congenital heart defects rely on large animal studies.

Genetic manipulation of mouse, zebrafish, and chicken embryos has all been used to create in vivo models to study the development and genetic basis of congenital heart defects [48, 49]. A zebrafish model found that heterozygous missense mutations of the *NFATC1* gene, a regulator of cardiac development, led to defects in cardiac looping and AV canal formation during cardiac development [50]. Transchromosomic mouse models developed by Kazuki et al. and Dunlevy et al. to simulate human Trisomy 21 have demonstrated robust Down syndrome features, including varying degrees of AVSD and other associated congenital heart defects [51, 52]. Another study by Tasaka et al. demonstrated that administration of bisdiamine—N, N-his (dichloroacetyl)‐1, 8‐octamethylenediamine—to pregnant rats led to an incidence rate of fetal AVSD over 50% of the time [53]. The AVSD morphology from this model was complete AVSDs with either truncus arteriosus of tetralogy of Fallot. Notably, these models are not amenable to surgical repair and modeling simulation due to size and reproducibility.

A canine model from 1981 to simulate complete AVSD repair was created by making incisions at the base of the mitral valves, and comparing outcomes based on the use of pledgetted stitches and spacing of stitches [54]. This model found that pledgetted stitches should be used in complete AVSD repairs, or non-pledgetted sutures should have closely spaced, deep bites. Despite this old study, robust in vivo large animal platforms for AVSD valves have not been reported in recent literature. Alteration of an animal’s native valve (e.g., incising the mitral valve to create a cleft mitral valve) does not represent the true pathophysiology of AVSDs as understood currently and creation of more complex valve geometries in conjunction with an atrial or ventricular septal defect remains a significant challenge without additional development work.

## Discussion

There is a great need for reliable scientific models to rigorously study and compare treatment approaches to AVSD patients. Different modeling platforms—in silico, in vitro, and in vivo—could provide valuable insight to improve the quality of care for these patients. This systematic review summarized the current landscape of modeling platforms to study congenital valve diseases (Table 1). Notably, we found a paucity of work specifically in the area of modeling AVSD valves and subsequent surgical repair. Therefore, we sought to summarize the leading possible sources for AV valve modeling data to understand this complex disease process in the future and stimulate further investigation in the field.

**Table 1:** Overview of Modeling Platforms to Study Abnormal Congenital Heart Valves

Year	Authors	Modeling type	Subtype / modality	Main pathology studied
2015	Salinas M, Ramaswamy S	In Silico	Computational Fluid Dynamics	Heart Valve Fluid Models
2024	An K et al.			Bicuspid Aortic Valve
2018	Park J, Bonde P		Finite Element Analysis	Mitral Valve in Dilated Cardiomyopathy
2020	Nappi F et al.			Pulmonary Autograft in Ross
2024	Shen X et al.			Bicuspid Aortic Valve
2025	Ju J et al.			Bicuspid Aortic Valve
2021	Emendi M et al.		Fluid Structure Interaction	Bicuspid Aortic Valve
2023	Morany A et al.			Bicuspid Aortic Valve
2023	Helbock RT et al.			Bicuspid Aortic Valve
2024	Qin T et al.			Bicuspid Aortic Valve
2024	Kaiser AD et al.			Bicuspid Aortic Valve
2022	Nam HH et al.		Visualization	Unrepaired Complete AV Canal
2025	Ponzoni M et al.			Complex Congenital Heart Disease
2015	Olivieri LJ et al.	In Vitro	3D Printing	Ventricular Septal Defects, Aortic Valve Leaks
2017	Ilina A et al.			Congenital Heart Valves
2017	Yoo SJ et al.			Congenital Heart Disease
2019	Vettukattil JJ et al.			Congenital Heart Disease
2020	Perens G et al.			Congenital Heart Disease
2020	Garg R, Zahn EM			Valvular Heart Disease
2020	Van Arsdell GS et al.			Congenital Heart Disease
2021	Yıldız O et al.			Congenital Heart Disease
2022	Seckeler MD et al.			Congenital Heart Disease
2022	Yoo SJ et al.			Atrioventricular Connections
2023	Mohanadas HP et al.			Cardiovascular Disease
2024	Kolcz J et al.			MAPCA Unifocalization
2024	Shiraishi I et al.			Congenital Heart Disease
2024	Ryan JR et al.			Congenital Heart Disease
2024	Lobbestael AJ et al.			Adult Congenital Heart Disease
2024	Shiraishi I et al.			Congenital Heart Disease
2025	Erdem S et al.			Congenital Heart Disease
2014	Juraszek A et al.		Flow Loop	Bicuspid Aortic Valve
2014	Vismara R et al.			Quadricuspid Aortic Valve
2022	Anam SB et al.			Bicuspid Aortic Valve
2020	Boone N et al.			Hypoplastic Left Heart Syndrome
2021	Zhu Y et al.			Bicuspid Aortic Valve
2021	Arimura S et al.			Bicuspid Aortic Valve
2022	Zhu Y et al.			Bicuspid Aortic Valve
2022	Romagnoni C et al.			Bicuspid Aortic Valve
2024	Kidambi S et al.			Single-Ventricle AV Valves
2024	Choi PS et al.			Bicuspid Aortic Valve
2017	Kheradvar A et al.	In Vivo	Review on Animal Models	Valvular Disease
2024	Kelly RG			Congenital Heart Defects
1981	Katz NM et al.		Canine Model	Atrioventricular Valves
1993	Tasaka H et al.		Murine Model	Atrioventricular Septal Defects
2010	Dunlevy L et al.			Atrioventricular Septal Defects (Down syndrome)
2020	Kazuki Y et al.			Atrioventricular Septal Defects (Down syndrome)
2018	Ferese R et al.		Zebrafish Model	Atrioventricular Septal Defects

 In silico modeling has gained traction in recent years, given advancements in computational capabilities and the availability of patient-specific imaging data. These advancements have allowed for maintenance of high fidelity in both hemodynamic flow and valvular biomechanics, a contrast to many previous models where only one aspect is accurately modeled [55]. Computational simulations are also valuable in their relative resource effectiveness and ease of replicating studies or customizing parameters. These factors make in silico modeling excellent for exploratory studies where novel repair strategies or anatomical variants can be quickly tested and iterated upon. However, computational simulations are built upon various underlying assumptions, so validation with other modeling platforms will be needed.

 In vitro modeling is currently the most common modality used by various institutions for anatomic visualization and spatial relations, specifically with 3D printed models to help with surgical training/education, diagnosis of complex anatomies, and preoperative planning. While 3D models are useful in these aspects, they do not mimic the elastomeric properties of functional heart valves and cannot be validated against in vivo or in silico functional hemodynamic data. While 3D models may help with surgical education and basic preoperative visualization, they do not offer the ability to study and compare outcomes of different surgical strategies. In vitro modeling using a pulse-duplicator flow loop is vital to address this need and allow for replicability and control over the valve testing environment. Studies using pulse duplicators have generated tremendous new knowledge in the adult heart valve space and have begun providing valuable insights into repair strategies for select congenital heart valve diseases. Specifically, existing models have been able to study the hemodynamics of valves with uniquely abnormal morphology, which will be critical when studying AVSD valves given the wide spectrum of disease severity. In addition, existing flow loops have allowed for the study and comparison of different valve repair techniques and have identified important parameters in aortic valve repair such as the free edge length to aortic diameter ratio. This could have implications for AVSD, as current repair strategies, such as the single-patch, modified single-patch, and two-patch techniques, have primarily been studied via clinical observation instead of a controlled laboratory setting. Other aspects of AVSD valve repair such as annuloplasty techniques and neochordal implantation can also be studied, and are questions that flow loops targeting adult mitral or aortic valves have already been applied to for testing. Given the current lack of reproducible platform to study AVSD valve repair strategies, we believe that in vitro testing using a flow loop represents a crucial next step towards a robust platform specifically for AVSD. However, challenges may arise when designing a platform to test morphologically complex AVSD valves. Primarily, abnormal AVSD valves that match patient-specific anatomy and biomechanical properties will need to be created with high fidelity, which will be more challenging than previous models, where simple modifications could be made to healthy valves (such as joining the left-right cusp to create a bicuspid aortic valve). Emerging fabrication technologies, such as double-component deposition electrospinning, which can recreate native-like heart valve structure and function, may be applied in these circumstances as the study material [56, 57]. Secondly, the valve holder must be adaptable to various valve sizes and shapes. It is also possible that a customized valve holder will be needed for each unique valve morphology tested. All these different valve holders must also be able to be securely incorporated into the flow loop. Furthermore, customizing the connections between the atrium, valve holder, ventricle, and outflow tract becomes challenging when flow may not be limited to a single direction in cases of complete AVSDs. Challenges associated with circulatory loop design, such as engineering components that could create artifacts when conducting in vitro experiments, highlight the need for multidisciplinary collaboration to ensure the in vitro platform generates physiologically accurate data [58]. 

In vivo, large animal models remain the highest fidelity platform to study valvular hemodynamics and repair strategies at physiological conditions. However, this technology is still in its nascent stages, with AVSD models limited to small animals, of which the only mammalian model is a mouse models. Overall, the development of in vivo animal models for studying AVSD valves currently face significant challenges, but could be a valuable tool to study the physiological effects and repair strategies of AVSD valves in the future.

A question that arose during the course of this review was the utility of these particular modeling platforms to the active clinician. In silico hemodynamic and biomechanical modeling are still primarily laboratory-based experimental models due to computing complexity, inability to cross-validate with in vitro or in vivo modeling, and need for extended time to construct. With the advancement of cross-sectional and 3D clinical imaging modalities, 3D geometric modeling and thus 3D printing are readily attainable with little lead time in several clinical institutions; however, the utility beyond pathology visualization is limited. Constructing custom flow loops with biomimetic AVSD valve models is also currently a multi-step process that takes weeks to months, so may not be readily usable to the clinician. With additional ongoing commercial development of these technologies, rapid printing of patient-specific valve replicas may become more widespread for clinical use. Ideally, a more complete FSI in silico model could be produced for clinical use including simulated valve repair strategies so real-time modeling and feedback could optimize surgical repair strategy. The creation of this end-result ideal model will rely on the successful integration of all three phases of modeling (in silico, in vitro, in vivo) to completely inform on outcomes from procedural manipulations of AVSD valves.

While each modeling modality has unique pros and cons, a thorough platform would require data from multiple different study designs as well as correlation with clinical data. For example, the study by Nappi et al. combined both a large animal study and computational simulation to study the benefits and potential modes of failure of the pulmonary autograft after the Ross procedure [12]. Anam et al. similarly created both in silico and in vitro models to study paravalvular leak after transcatheter replacement of bicuspid aortic valves, and demonstrated that the findings not only were in agreement between the modeling platforms, they also matched the actual patient clinical data [42]. These examples point to the strengths of cross-validating modeling platforms and the need to compare findings from simulations to real patient data to ensure the accuracy of simulations. It is our analysis from this review article that these cross-validated platforms provide the most rigorous performance metric when dealing not only with the pathophysiology of an AVSD valve but allowing understanding and testing of actual surgical manipulation strategies for durability. The use of multiple cross-validated platforms (select in silico, in vitro, and eventually in vivo) together to create a feedback loop will be the most reliable way to understand and predict the behavior of AVSD valves after repair. While it seem most likely that eventually advanced FSI models will eventually be able to handle modeling of surgical repairs, the feedback to build and confirm these models from the in vitro and in vivo environment will be critical.

## Conclusion

Currently, there is no robust platform designed to study the complex biomechanics and hemodynamics of the various valve morphologies observed in AVSD. While clinical outcomes after repair have improved over the decades, there remains a significant risk for reoperation. In vitro and in silico models have shown great potential as platforms to rigorously quantify valvular diseases and test repair strategies and are beginning to be employed for complex congenital valves. While in vivo modelling of AVSD currently face significant barriers, continued advancements may make this option a reality in the future. Collaborative and creative methods of leveraging the strengths and weaknesses of each modality can allow investigators to thoroughly expand understanding and generate unique insights into AVSD pathology and durable repair strategies. In conclusion, interdisciplinary efforts towards creating high-fidelity, customizable AVSD valve modelling platforms can transform the paradigm of how AVSD is studied and propel this field towards a future of personalized repair strategies catering to each patient’s unique anatomy and physiology.

## Supplementary Information

Below is the link to the electronic supplementary material.

Supplementary Material 1

Supplementary Material 2

Supplementary Material 3

## Data Availability

Data sharing not applicable to this article as no datasets were generated or analyzed during the current study.

## Abbreviations

AV: atrioventricular
AVSD: atrioventricular septal defects
CT: computed tomography
LAVVR: left atrioventricular valve regurgitation
MAPCA: major aortopulmonary collateral arteries
MRI: magnetic resonance imaging
