# Supplementary material for: A Systematic Review of Modeling Platforms for Atrioventricular Valves in Atrioventricular Septal Defects
Source: J Cardiovasc Transl Res. 2026 Jun 18;19(1):73. doi: 10.1007/s12265-026-10799-z (PMC13279604; doi:10.1007/s12265-026-10799-z)
Supplement: Supplementary file 3 — Supplementary Material 3 [file 12265_2026_10799_MOESM3_ESM.docx]

Search Strategy

On PubMed, our search strategy included the Medical Subject Heading “heart defects, congenital”; AND text words "atrioventricular canal defect" OR "atrioventricular septal defect" OR "common atrioventricular canal" OR "complete atrioventricular canal" OR "complete atrioventricular septal defect" OR "partial atrioventricular canal" OR "atrioventricular valve" OR "valve disease” OR "valvular disease" OR "abnormal valve"; AND text words "in vivo" OR "in vitro" OR "ex vivo" OR "simulate" OR "simulation " OR "in silico" OR "computational" OR "model" OR "3D printing".

On Scopus, our search strategy included searches within the article title, abstract, and keywords for: (“atrioventricular canal defect" OR "atrioventricular septal defect" OR "common atrioventricular canal" OR "complete atrioventricular canal" OR "complete atrioventricular septal defect" OR "partial atrioventricular canal" OR "valve disease" OR "valvular disease" OR "abnormal valve") **AND** ("in vivo" OR "in vitro" OR "ex vivo" OR "simulate" OR "simulation " OR "in silico" OR "computational" OR "model" OR "3D printing") **AND** "congenital"

On Embase, our search included both EMTREE terms and free text in the title, abstract, or keywords. Our final search was: ('atrioventricular septal defect'/exp OR 'atrioventricular canal defect'/exp OR 'valve disease' OR 'atrioventricular canal defect':ti,ab,kw OR 'atrioventricular septal defect':ti,ab,kw OR 'common atrioventricular canal':ti,ab,kw OR 'complete atrioventricular canal':ti,ab,kw OR 'complete atrioventricular septal defect':ti,ab,kw OR 'partial atrioventricular canal':ti,ab,kw OR 'valve disease':ti,ab,kw OR 'valvular disease':ti,ab,kw OR 'abnormal valve':ti,ab,kw) AND ('congenital disorder'/exp OR congenital:ti,ab,kw) AND ('in vivo':ti,ab,kw OR 'in vitro':ti,ab,kw OR 'ex vivo':ti,ab,kw OR simulate:ti,ab,kw OR simulation:ti,ab,kw OR 'in silico':ti,ab,kw OR computational:ti,ab,kw OR model:ti,ab,kw OR '3d printing':ti,ab,kw OR 'additive manufacturing':ti,ab,kw). EMTREE terms were denoted with /exp in the search, and free text in the title, abstract, or keywords were shown as :ti,ab,kw in the search.
